# P-1865. Impact of Outpatient Parenteral Antimicrobial Therapy (OPAT) Service on Clinical Outcomes: A Single Health System Retrospective Cohort Study

**DOI:** 10.1093/ofid/ofaf695.2034

**Published:** 2026-01-11

**Authors:** Saipriya Gadiraju, Nikunj M Vyas, Shereef N Ali, Alissa Werzen

**Affiliations:** Jefferson Health East Region, Hartford, Connecticut; Jefferson Health - New Jersey, Marlton, NJ, New Jersey; Jefferson Health New Jersey, Cherry Hill, New Jersey; Jefferson Health New Jersey, Cherry Hill, New Jersey

## Abstract

**Background:**

Recent studies indicate benefits of OPAT include shorter hospital stays, decreased costs, and improved patient quality of life. This study's purpose was to show the clinical impact of an OPAT program on patients being discharged on IV antimicrobials.

**Table 1:**
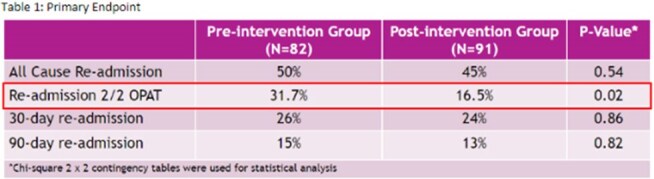
Primary Endpoint * Chi-square 2X2 contingency tables were used for statistical analysis

**Figure 1:**
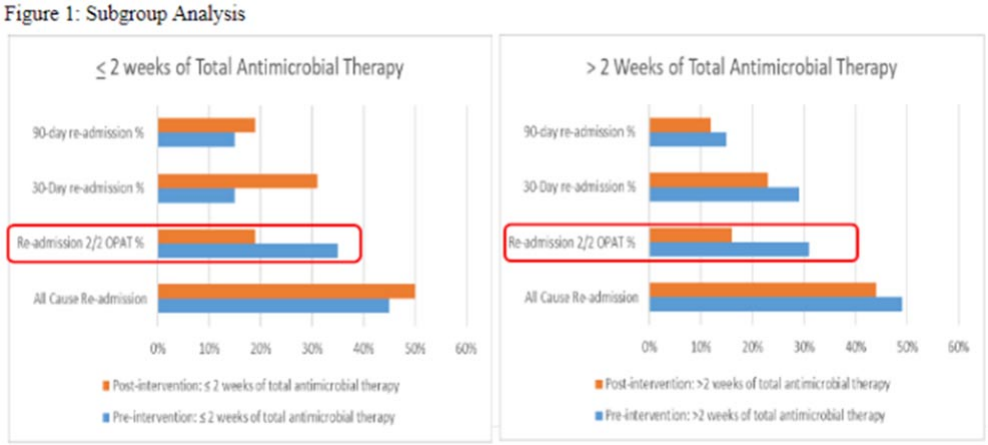
Subgroup Analysis

**Methods:**

This was an IRB approved retrospective chart review of adult patients admitted between January 1st to December 31st, 2023. Inclusion criteria was patients at least 18 years of age who were discharged on intravenous antimicrobials for >7 days of therapy remaining at the time of discharge. Participants were excluded if they received OPAT without an ID consult or were transferred to a different acute care facility. The primary outcome of the study was to evaluate the impact of OPAT program on 30 and 90 day readmission as well as OPAT related readmission. Secondary outcomes were the treatment success rate at completion of therapy and compliance of outpatient ID follow up.

**Figure 2:**
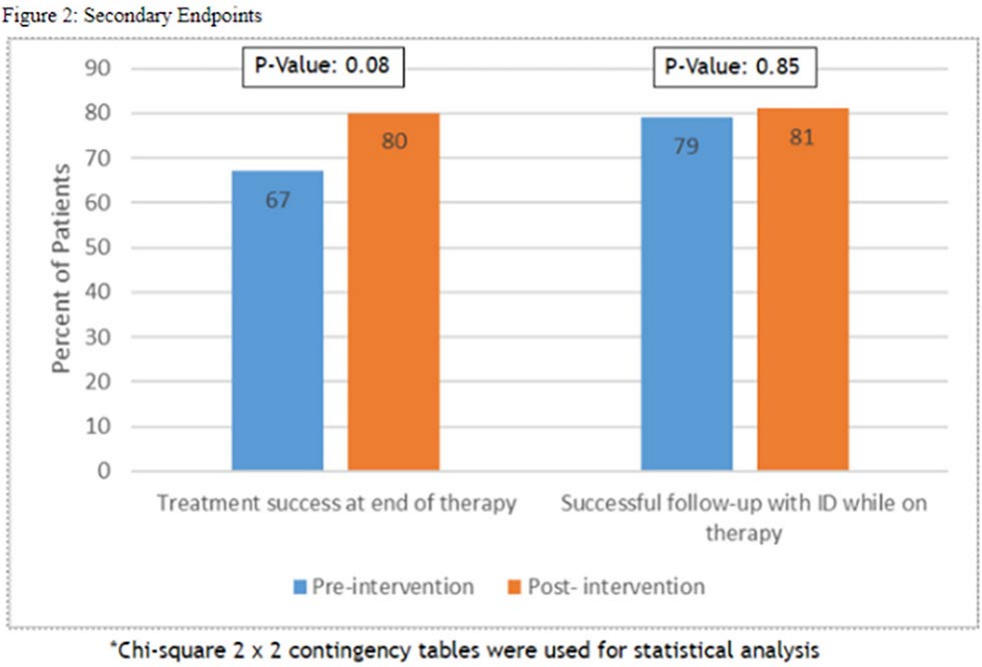
Secondary Endpoints

**Results:**

A total of 173 patients were included in the final analysis with 82 patients in the pre-intervention group (PRIG) and 91 patients in the post intervention group (POIG). Demographic findings between patients in both groups were similar. The most common indications were bone and joint infection followed by bacteremia and discharge to home with home infusion was the most common disposition. S*taphylococcus aureus* was the most common pathogen identified in both groups. Patients in the POIG were less likely to experience OPAT related readmissions (31.7% vs 16.5%, P=0.02). There was no difference in all-cause, 30 and 90 readmission between two groups. In the subgroup analysis, OPAT related readmissions were more common in patients who needed >2 weeks of OPAT compared to < 2 weeks (27.7% vs 22.7%, P=NS). There were trends towards higher treatment success with the POIG compared to the PRIG (67% vs 80%, P=0.08). Rates of outpatient follow-up with ID provider were similar between groups (79% vs 81%, P=0.85).

**Conclusion:**

Implementation of OPAT care team service shows a promising decrease in OPAT related re-admission. Implementation of an OPAT care team has the potential to lead to improved clinical success. A study with a larger sample size should be done to confirm these results and draw further conclusions.

**Disclosures:**

All Authors: No reported disclosures

